# Predicting early failure of quantum cascade lasers during accelerated burn-in testing using machine learning

**DOI:** 10.1038/s41598-022-13303-0

**Published:** 2022-06-02

**Authors:** Cagri Aydinkarahaliloglu, Shashank Jatar, Xiaojun Wang, Mary Fong, Vijay Gupta, Mariano Troccoli, Anthony J. Hoffman

**Affiliations:** 1 Department of Electrical Engineering, University of Notre Dame, Notre Dame, IN 46556 USA; 2 AdTech Photonics, 18007 Cortney Court, City of Industry, CA 91748 USA; 3 Evolution Photonics Inc, 931 E Walnut St, Pasadena, CA 91106 USA

**Keywords:** Lasers, LEDs and light sources, Computer science

## Abstract

Device life time is a significant consideration in the cost of ownership of quantum cascade lasers (QCLs). The life time of QCLs beyond an initial burn-in period has been studied previously; however, little attention has been given to predicting premature device failure where the device fails within several hundred hours of operation. Here, we demonstrate how standard electrical and optical device measurements obtained during an accelerated burn-in process can be used in a simple support vector machine to predict premature failure with high confidence. For every QCL that fails, at least one of the measurements is classified as belonging to a device that will fail prematurely—as much as 200 h before the actual failure of the device. Furthermore, for devices that are operational at the end of the burn-in process, the algorithm correctly classifies all the measurements. This work will influence future device analysis and could lead to insights on the physical mechanisms of premature failure in QCLs.

## Introduction

Quantum cascade lasers (QCLs) are electrically-injected semiconductor lasers that produce photons in the mid-infrared (λ ~ 3–30 μm) and terahertz (λ ~ 30 μm–3 mm) portions of the electromagnetic spectrum. In contrast to diode lasers, which produce photons via electron–hole recombination across a semiconductor bandgap, QCLs generate photons via intersubband transitions—electronic transitions between the quantized energy levels of quantum well heterostructures^1^. Many of the characteristics of QCLs can be tailored via the quantum design of the heterostructure, and recent progress has resulted in expanded spectral coverage and significant improvements in their overall performance^2–9^. These improvements have enabled or improved the use of QCLs in a growing number of applications in trace gas sensing, imaging, and defense^10–12^. More recently, QCLs have been shown to support self-starting frequency combs, which will further increase their utility and open new opportunities for the devices^13–15^. However, their broad use in other applications, particularly those that involve integration into complex systems, can be limited due to concerns over premature device failure. Such premature device failure has been observed in practice as discussed in more detail below, is ill-understood from a mechanistic point of view, and drastically increases the cost of ownership of QCLs.

There have been several studies on the life time of QCLs under various testing conditions^16–19^. The average life time of QCLs has been estimated as 809 thousand hours when operated at a heat sink temperature of 25 °C^18^. A major challenge in estimating the life time of QCLs is the low failure rate of the device past an initial operating period. Indeed, in much of the previous life time studies, the tested QCLs exhibit two timescales for failure: within several hundred hours of operation or after thousands of hours of operation; those studies focus on lasers that survive the initial operating period^19–21^. Premature failure refers to failures within the shorter timescale. Identifying QCLs that fail prematurely is typically accomplished using laser burn-in processes where the performance of the QCLs under test are monitored over time. This requires establishing a burn-in process that is sufficiently long in duration to observe the premature failure of devices. While longer burn-in times can increase the likelihood of observing premature failure, doing so can be costly and time-consuming. Additionally, it is not always possible to identify lasers that will fail prematurely after the conclusion of the initial burn-in period as the mechanisms for premature failure and its manifestation on the operational characteristics of the QCLs are not known.

An alternative approach to existing methods is to develop decision algorithms that use measured optical and electrical characteristics of QCLs to *predict* which lasers are likely to fail. Since the physical mechanisms leading to device failure are yet unknown, we do not know precisely which characteristics are predictive of device failure and in what regimes of values. However, as we show in this paper, a machine learning approach can identify relevant signatures of failures in these characteristics and reliably predict imminent device failure up to 200 h in advance. This is an interesting and a novel finding because it enables fast and reliable identification of QCLs that are likely to fail, reducing the resources expended on the entire burn-in process.

Machine learning (ML) is the study of improving systems automatically through experience^22^. ML has been used to determine or predict a particular outcome given specified input data in various applications from computer vision to speech recognition to natural language processing^23–25^. There are a variety of approaches to ML. In supervised ML, the objective is to develop a mapping function between the input and output data based on example input–output pairs. Support vector machines (SVMs) are a powerful means to implement this supervised ML paradigm. The objective of a SVM is to build and train a model with two classes using a set of *training* data consisting of a set of input data labeled with the correct class to which the data belong. Its advantages include not requiring as much data as other approaches such as recurrent neural networks, and the binary classification provided by the SVM is ideal for identifying operational and defective devices. The trained model is used to assign input data with an unknown class label to the correct class. During training, the SVM maps the training data to points in an N-dimensional space, where the dimensions are used to represent features of the dataset. Since the correct class for each training data point is known, the SVM can then calculate a hyperplane in the N-dimensional space that maximizes the distance between the set of data points belonging to one class from the set belonging to the other class. Once training is complete, new data is mapped onto the same N-dimensional space and classified using the hyperplane that has been learned^26,27^. Successful use of SVMs has been demonstrated in various applications, including milling chatter detection^28^, recognizing complex machining conditions^29^, gene selection for cancer classification^30^, and fault detection^31,32^.

In this work, we use a SVM to identify devices that will fail prematurely using conventional measurements of the electrical and optical characteristics of QCLs obtained during accelerated burn-in testing^33^. We develop an approach for parametrizing the electrical and optical measurements and use those results to train and test a SVM model. The trained model predicts failure as much as 200 h before the observed premature failure of a QCL. Importantly, the SVM never identifies a device that does not fail as one that will. Such an SVM can enable a significant reduction in QCL manufacturing costs by identifying devices that are likely to experience premature failure early in the burn-in process, reducing the time spent on burn-in testing for those devices. Additionally, the fact that failure can be predicted using observations up to 200 h in advance will enable future research into the mechanisms of premature failure in QCLs.

## Methods

### Experimental setup

**Figure 1:**
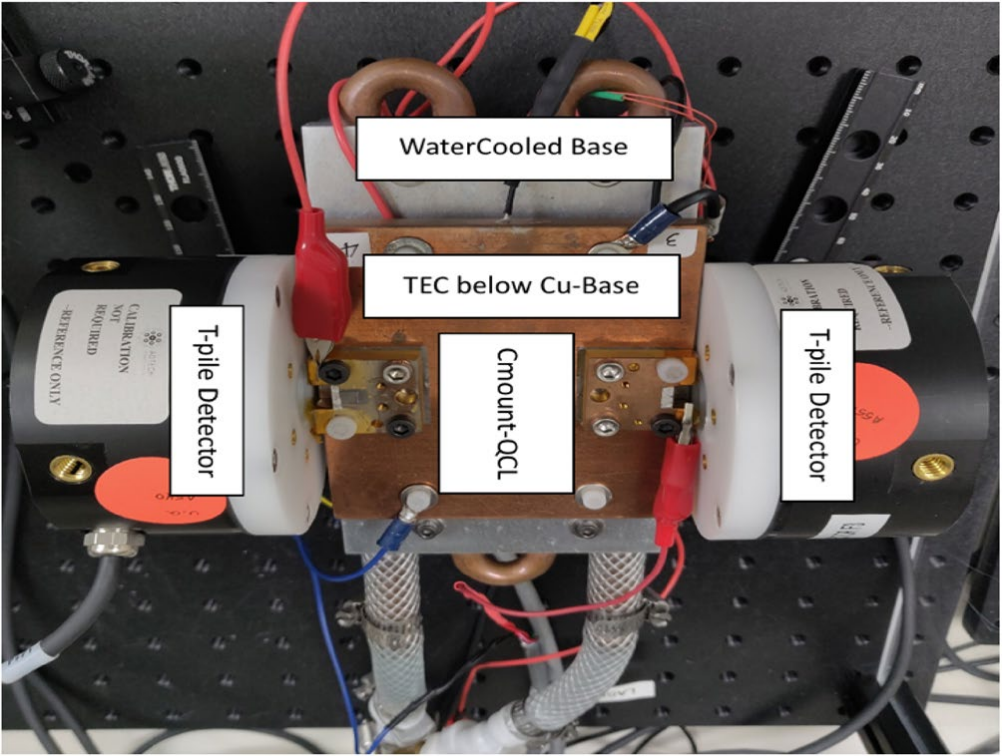
QCL Mount with integrated water-cooled TEC for high temperature accelerated lifetime testing.

We test nine high-performance, Fabry–Perot QCLs using an accelerated burn-in process where the lasers are operated in continuous wave (CW) mode at an elevated heat sink temperature. The QCLs are from different portions of a single epitaxial wafer grown by metal organic chemical vapor deposition and therefore share the same multiple quantum well heterostructure design. The lasers are processed as buried heterostructures using InP regrowth and mounted epitaxial-side down on CuW C-mounts for efficient heat extraction. The QCLs are all 5 mm long, but their ridge width varies. Two identical testing stages are used for the accelerated burn-in testing. Each stage uses a temperature-controlled thermoelectric cooler mounted on a water-cooled base. The stages are both equipped with two thermopiles; thus, four QCLs (two per stage) can undergo the accelerated burn-in process simultaneously as depicted in Fig. 1.

The planned burn-in time for seven of the QCLs is 100 h, which is typical in burn-in experiments. However, there are differences in the actual burn-in lengths due to starting and stopping the burn-in process manually. Additionally, two of the devices fail before the end of the planned burn-in procedure and two other QCLs undergo longer accelerated burn-in testing to further validate the SVM-based classifier. In practice, the testing for a QCL would end as soon as it is classified as a device likely to fail by the SVM or the end of the burn-in period is achieved. For the two devices that undergo extended burn-in testing, the interval between CW LIV measurements is increased and ranges from 10 to 48 h between measurements. During the accelerated burn-in process, the QCLs are operated in CW mode at 80% of their maximum CW optical power at a heat sink temperature of 313 K. Measurements of the CW current, voltage, and optical power are recorded every minute. Here, these frequent measurements are only used to determine when a device fails, which is indicated by measuring no output optical power from the biased device. Continuous wave light–current-voltage (LIV) measurements are performed approximately every 2.5 h where the current, voltage, and output optical power are measured as the CW current is swept in 10 mA steps from zero to the operating point (80% of the maximum output power).

**Figure 2:**
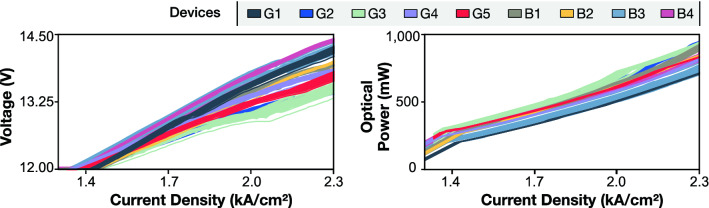
Current density versus voltage (left) and current density versus optical power (right) characteristics for the nine devices. After burn-in, the devices that were still functional are denoted with G1, G2, G3, G4 and G5, whereas defective devices are denoted as B1, B2, B3 and B4. Device condition identification by eye is not apparent.

Figure 2 shows every measured LIV for all nine QCLs obtained during the accelerated burn-in processes. The devices labeled G_N_ (N = 1…5) indicate QCLs that are operational at the end of the burn-in testing. The devices labeled B_N_ (N = 1…4) indicate QCLs that failed during the burn-in testing. In Fig. 2, we omit measurements after device failure for devices B1 to B4. This data is also excluded when training and testing the SVM. Importantly, for the measurements shown in Fig. 2, there is no easily apparent distinction between the LIVs of devices that remain operational and devices that fail. However, we will show that incorporating the LIV measurements into SVMs can predict premature failure of the QCLs with very high accuracy.

We incorporate the LIV measurements into a SVM by extracting features from the measurements that express the significant characteristics of the data. The parameters are extracted automatically using custom software that generates a feature matrix for each LIV. Examples of extracted features include the wall-plug efficiency (WPE) at maximum optical power; laser threshold current density; applied voltage at lasing threshold; maximum output optical power; slope of optical power versus current density at several operating points; and differential resistance (slope of voltage versus current density) at several operating points. In total, we extract 28 features describing each LIV measurement. All of the features are listed in Table 1. Here, *V* is the voltage; *P* is the measured optical power, *P*_*max*_ is the maximum optical power measured from one facet, *J* is the current density, and *R* is the differential resistance.

**Table 1:** Features used in the SVM model.

At P_th_	At 1.1J_th_	At P_max_	Minimum	Maximum	Mean	Standard deviation
V, J	V	V				
$dP/dJ$	$dP/dJ$	$dP/dJ$	$dP/d$ J	$dP/d$ J	$dP/dJ$	$dP/d$ J
$dV/d$ J	$dV/d$ J	$dV/d$ J	$dV/d$ J	$dV/d$ J	$dV/dJ$	$dV/d$ J
$dR/dJ$	$dR/d$ J	$dR/dJ$	$dR/d$ J	$dR/d$ J	$dR/dJ$	$dR/d$ J
R			R	R		

### Training and testing

We used the Python package scikit-learn for training and testing the SVM. To predict premature device failure, we use the 28 features in a SVM with a radial basis function (RBF-SVM) kernel as the overall accuracy and number of false negatives was superior compared to the linear SVM. The training and testing of the model consist of two stages. In the initial stage, the model is trained and tested with data from the first set of devices with planned burn-in times of 150 h *(G*_*i*_*; i* = *1…5, B*_*j*_*; j* = *1,2)*. During training and testing, the LIVs of a single device are categorized according to the operational status of the device at the end of the burn-in period. For a device that fails during testing, all the LIVs are categorized as belonging to a failed device. Likewise, for a device that is operational at the end of testing, all the LIVs for that device are assumed to come from an operational device. For the set of QCLs part of the 150-h burn-in process, there are 444 individual LIV measurements in total. 95 of the LIVs are from devices that fail during testing. The remaining 349 LIV measurements are from devices that are operational at the end of testing.

Hyperparameters are parameters that control the learning process. The RBF-SVM has two hyperparameters, *Γ* and *C*, that are adjusted during training to achieve high classification accuracy. The *Γ* hyperparameter controls the amount of influence of a single training point whereas *C*, adds a penalty to each misclassified point^34^. To tune these hyperparameters, we employ a grid-search algorithm, where we vary the parameters between 10^–3^ to 10^3^ with an adaptive step size, yielding a parameter matrix of 72 rows by 72 columns. For each pair of hyperparameters, we calculate the accuracy of the SVM. To avoid overfitting to a particular set of training data, we repeat this process for 10 different sets of training data. Each of the 10 training data sets consists of LIVs from four operational devices and one failed device. A score is assigned to each pair of hyperparameters, by averaging the classification accuracy scores of the 10 cases. In the end, the SVM with the hyperparameter pair yielding the highest score is selected for use in the model. We emphasize that even though we use different combinations of devices in the training and test sets, we never mix the two sets in any experiment. In other words, the SVM is never trained on the devices in the test set. When using the RBF-SVM to classify the LIVs for the two devices that underwent the longer burn in process, we train the SVM using *(G*_*i*_*; i* = *1…5, B*_*j*_*; j* = *1,2)*. The RBF-SVM uses the same values for the hyperparameters that were determined previously.

## Results and discussion

**Figure 3:**
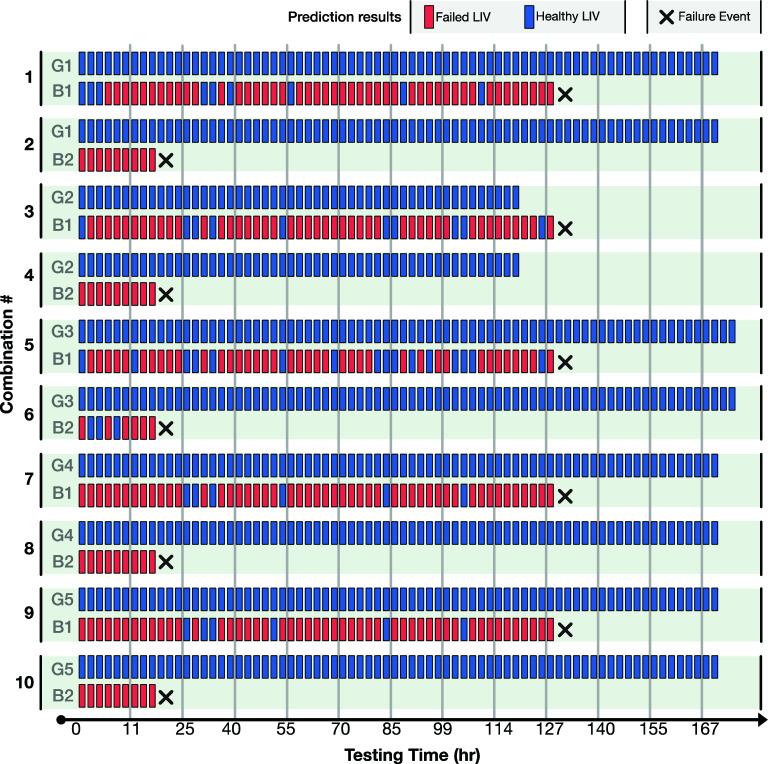
SVM testing results for the first stage of experiments. Rectangles represent measurement events at different time points, while their color represent the model’s prediction. The failure event is marked with a black cross. The SVM model predicts failure for all of the defective devices up to 127 h prior to the failure event.

To evaluate the SVM based classifier, we use every possible combination of training and testing data sets, 10 combinations in total. Figure 3 shows the predictions of the model for different combinations of test data. Here, for each combination, the algorithm was trained on the LIVs from the remaining five devices (four operational devices and one failed device) and tested using the indicated operational (G) and failed (B) devices. For example, for Combination 1, LIVs from G2, G3, G4, G5 and B2 are used for training the SVM and LIVs from devices G1 and B1 are used for testing the SVM. Each colored rectangle represents a prediction made by the SVM for the LIV measured at the indicated time. The time of the QCL failure is indicated with a black cross.

As shown in Fig. 3, the model identifies healthy devices with 100% accuracy for every test combination, encompassing over 1500 h of measurements for the operational devices. Furthermore, for each device that fails during the burn-in testing, the SVM categorizes multiple LIVs as belonging to a failed device many measurements (hours) before the actual failure of the QCL. In some instances, the first measured LIV is categorized as belonging to a failed device. With no false negatives (identifying an LIV from an operational device as belonging to a failed device) observed, high confidence can be assigned to predicting devices will fail even from a single LIV that is categorized as coming from a failed device.

**Figure 4:**
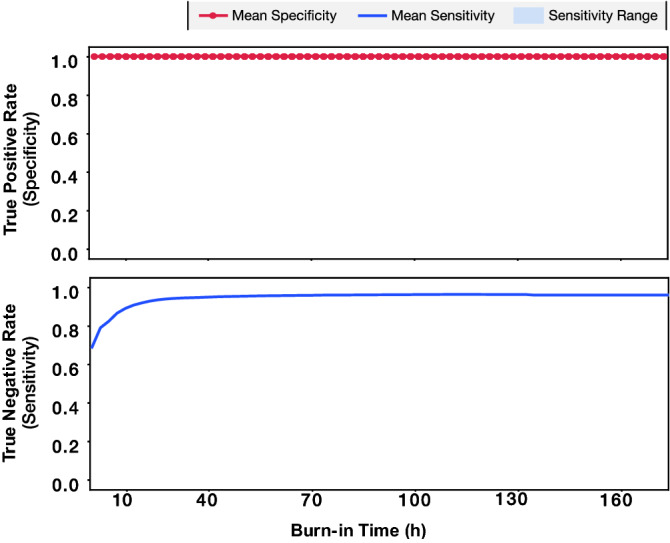
Calculated mean specificity and sensitivity with respect to burn-in time. The blue shaded region indicates the sensitivity range across the 10 different combinations of training and testing data sets. The mean sensitivity of all 10 cases is plotted onto the shaded region as a solid line.

We use the output of the SVM to classify the QCL as healthy or failing. A device is classified as failing at a measurement interval if any of the LIV measurements up to that point in time are identified as belonging to a device that will fail (red boxes in Fig. 3). Otherwise, the device is classified as healthy. To evaluate the SVM based classifier, we calculate the specificity and sensitivity for each of the measurement intervals for all 10 combinations of training and testing datasets. The specificity, also known as true positive rate, is the ratio of the number of devices predicted as healthy over the total number of healthy devices, i.e., operational devices at the conclusion of the burn-in process. The sensitivity is the ratio of the number of devices predicted as failing over the total number of devices that fail during the burn-in process. For the seven QCLs scheduled for the shorter burn-in time, the average specificity is 100% and the average sensitivity for the 10 cases is 93.65% across all the hours. In Fig. 4, the change of sensitivity and specificity with respect to the burn-in time is depicted. A sensitivity greater than 80% is obtained after 8 h of burn-in time. Furthermore, for every combination of test and training data, the specificity is constant at 100%. For these QCLs, premature failure can be predicted as much as 129 h before the actual failure of the device.

**Figure 5:**
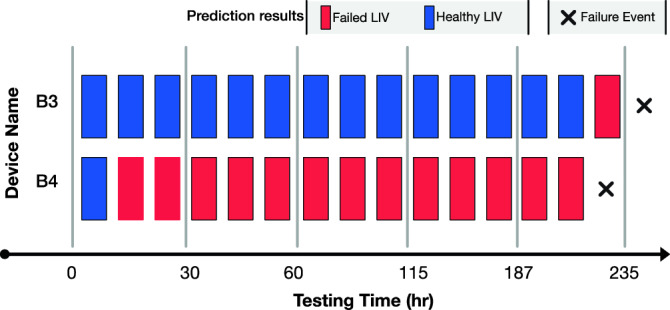
SVM testing results for the second stage of experiments. Rectangles represent measurement events at different time points, while their color represent the model’s prediction. The failure event is marked with a black cross.

Two of the devices were operated for longer burn-in periods, because device B4 was classified as a failed device 13 h into the burn-in testing. To further validate the SVM based classifier, we decided to run the tests until B4 fails, which occurred 200 h after the start of the burn-in testing. During this longer burn-in, the SVM-based classifier labeled B3 as a failed device as well; its eventual failure occurred after 235 h of testing. Figure 5 shows the results of classifying the measured LIVs from these devices. For device B3, the first LIV classified as belonging to a device that will fail occurs 13 h before the failure of the QCL. For device B4 however, the second measured LIV indicates that the device will fail prematurely, 200 h before the actual device failure. While the lead times between identifying the QCL as a device that will fail and the actual failure for these two devices are different, the SVM classifies at least one LIV as belonging to a failing device for each of the lasers. We speculate that the differences for these two devices could be related to the testing conditions. One possibility is that the device B3 was damaged during testing, for example from a brief power surge, and that damage ultimately resulted in the observed failure, whereas device B4 had underlying problems from the beginning of testing and would eventually exhibit premature failure.

Classifying LIVs using a SVM could have significant impact beyond predicting the premature failure of individual devices, and this is highlighted by the differences in the classification of the LIVs for devices B3 and B4. As more devices undergo the accelerated burn-in process and LIV measurements are classified, it will be possible to develop an understanding of the timescale for the failure process. In fact, the effectiveness of this approach using LIV measurements separated by three-hour intervals already gives important information about the timescale for the failure process. We expect that incorporating additional devices and measurements into this work will lead to further improvements in QCLs and new capabilities for filtering short-lived devices from commercial inventory.

## Conclusions and future work

This paper presents an accelerated burn-in process that incorporates a classifier based on a supervised learning algorithm for predicting premature device failure. The hyperparameters for the SVM-RBF are determined using multiple training data sets to minimize the possibility of overfitting. The presented findings indicate that operational QCLs are always identified correctly without any false negatives. In addition, all QCLs that fail have at least one LIV that indicates that it will fail. Collectively, this gives us high confidence that a LIV labeled as coming from a failing device indicates that there will be premature failure of that device. The SVM classifies some of the QCLs as devices that will exhibit premature failure based on the first LIV measurement, which can drastically reduce time spent on burn-in processes for devices that will fail. In the best case, this is 200 h before the actual failure.

Our approach to using a SVM-based classifier for predicting premature QCL failure can be extended to QCLs with different active region and waveguide designs. While the SVM may have to be retrained for different growths or wafers, the ability to identify lasers that are likely to fail prematurely will still have a large impact on production times. The generalization of SVMs across wafers and growths is an area of future research. Additionally, the ability of the existing classifier to predict premature failure up to 200 h before the actual device failure could provide direction for future research into the origins of premature failure in QCLs. Finally, the strategy of employing a SVM-based classifier for predicting premature failure can be incorporated into the QCL manufacturing process to decrease the overall cost of ownership.

## Data Availability

The data and code used to obtain the results presented in this paper and other findings of this study are available on GitHub: https://github.com/caydink/Predicting-Early-Failure-of-QCLs-using-Machine-Learning.git.
